# PML in the Brain: From Development to Degeneration

**DOI:** 10.3389/fonc.2013.00242

**Published:** 2013-09-17

**Authors:** Erica Korb, Steven Finkbeiner

**Affiliations:** ^1^Gladstone Institutes of Neurological Disease, San Francisco, CA, USA; ^2^Neuroscience Graduate Program, University of California, San Francisco, CA, USA; ^3^Biomedical Science Program, University of California, San Francisco, CA, USA; ^4^Taube-Koret Center for Neurodegenerative Disease Research, Gladstone Institutes, San Francisco, CA, USA; ^5^The Hellman Family Foundation Program in Alzheimer’s Disease Research, Gladstone Institutes, San Francisco, CA, USA; ^6^Department of Neurology, University of California, San Francisco, CA, USA; ^7^Department of Physiology, University of California, San Francisco, CA, USA

**Keywords:** circadian rhythms, synaptic plasticity, Arc, neurodegeneration, neural progenitor cells, neocortex development, SCN, PML

## Abstract

The promyelocytic leukemia (PML) protein is the main component of PML nuclear bodies, which have many functions in a wide range of cell types. Until recently, PML was not known to have a function in the nervous system or even be expressed in the brain. However, recent reports have changed that view. PML is found in neurons and functions in many aspects of the nervous system, including brain development, circadian rhythms, plasticity, and the response to proteins that cause neurodegenerative disorders. While the investigation of PML in the brain is still in its infancy, it promises to be a fascinating subject that will contribute to our understanding of the brain. Here we summarize what is known about PML expression and function in the brain and highlight both discrepancies in the field and areas that are particularly important to future research.

## Introduction

Promyelocytic leukemia originally identified from its role in acute PML. In this form of cancer, a chromosomal translocation results in a fusion protein containing PML and the retinoic acid receptor. The role of PML in cancer and several other cell types, particularly those involved in the immune response, has been extensively investigated (1–3). PML and the nuclear bodies they form carry out their functions through a variety of mechanisms from transcriptional regulation to protein degradation.

While the initial characterizations of PML uncovered no significant expression in the nervous system, more recent publications show it is expressed in dividing neural precursors and mature neurons. In these cells, it appears to regulate transcription, but the mechanisms are not yet understood.

In this review, we will describe the context in which PML is expressed in the nervous system and what is known about how this expression is regulated. We will also describe its functions in the nervous system and discuss the numerous areas that require more research for a complete understand of PML in the brain.

## PML Expression and Regulation in the Brain

Initial publications sought to characterize PML in neurons (4, 5) or neuron-like cells (6) and found little or no PML expression. However, upon closer examination, it is difficult to draw conclusions about PML in the adult brain from these findings.

The evidence that PML nuclear bodies are not found in mature neurons is not compelling. For example, one paper often cited in support of this position did not directly examine PML expression. The investigators characterized the staining pattern of an antibody resulting from mice injected with rat lymphocytes (5). While the antibody colocalized with PML nuclear bodies, it was not clear whether it recognized PML or one of the many proteins often found in the PML nuclear body that might not be expressed in neurons. Another commonly cited paper focused on NT2 cells, a neuronally committed human teratocarcinoma cell line (6). NT2 cells had low levels of PML expression, but when they were induced to differentiate into neuron-like cells, they expressed high levels of PML. It is difficult to draw conclusions about neurons from a carcinoma cell line, but the data presented in this paper do not suggest a lack of PML in neurons.

Regad et al. performed one of the first thorough examinations of PML in the brain, focusing on the developing neocortex (4). They found that PML is expressed in the ventricular zone at embryonic day 15. They also found expression in the ventricular zone but not in the cortical plate at postnatal days 0 and 7. However, a small number of cells expressed PML in the hippocampus in postnatal brains. The data characterize PML expression in the developing brain and also convincingly demonstrate that PML had a functional role in brain development (discussed in more detail below). Yet, these data did not rule out the possibility that PML is also expressed in mature neurons later in development or adulthood. In fact, the presence of PML in a subset of postnatal hippocampal cells suggests that it is expressed in fully differentiated neurons under specific conditions. This expression pattern suggests that PML is a highly regulated protein in the brain that is expressed in response to specific stimuli.

Other studies support the idea that PML is expressed in the brain in a highly regulated manner. PML RNA is present in the suprachiasmatic nucleus (SCN) of adult mice, which is involved in regulating circadian rhythms. Furthermore, the pattern of PML expression was highly regulated: PML protein levels peaked at specific times during the circadian cycle (7). In addition, PML was expressed in the hippocampus, cortex, cerebellum, and brain stem in adult mice (8).

One confounding issue with these studies is that brain tissue contains glial cells. Thus, these findings are not conclusive proof that PML is expressed in mature neurons. However, studies in neuronal culture systems provide additional support for PML expression in neurons. PML is expressed in neurons at low levels in a neuronal culture system that lacks glia. Upon neuronal stimulation, PML protein expression is significantly upregulated, as seen by western blot and live-cell imaging (9).

Other studies examined PML expression in the context of neurodegenerative disorders and stroke models. Using western blots and immunohistochemical analysis, Hayashi et al. found PML was upregulated in neurons in response to transient middle cerebral artery occlusion (10). PML is expressed in neurons in the presence of mutant polyglutamine proteins, such as ataxin (11, 12), and colocalizes with intranuclear inclusions in diseases, such as frontotemporal dementia (13). In some cases, PML is upregulated or PML nuclear bodies are rearranged or disrupted in response to these proteins and in the neurodegenerative diseases caused by these proteins (11, 12, 14–16). Interestingly, the cytokine interferon beta, which induces PML nuclear body formation in immune cells, also appears to increase PML expression in neurons in Purkinje cells in a culture model of spinocerebellar ataxia (11, 17).

**Figure 1:**
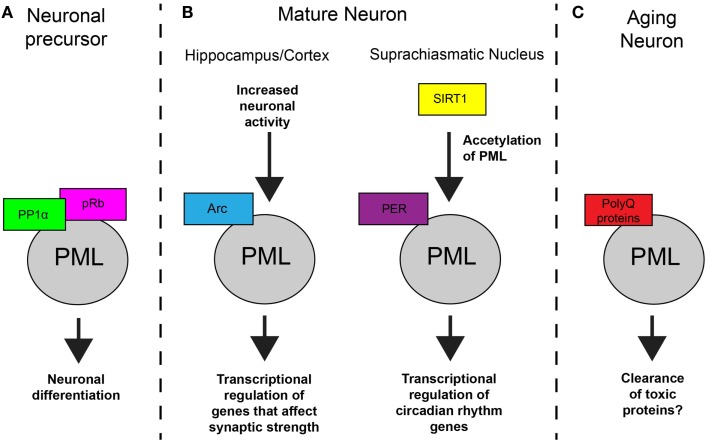
**Model of PML function in neurons**. **(A)** In NPCs, PML regulates subcellular localization of protein phosphatase 1α (PP1α) and retinoblastoma protein (pRB), which results in dephosphorylation of pRB and controls neuronal differentiation. **(B)** In mature neurons, PML is involved in synaptic plasticity and circadian rhythms. In response to increased activity, Arc mediates an increase in PML nuclear bodies which results in transcription regulation. In the SCN, SIRT1 regulations acetylation of PML which affects PER2 binding and transcription of genes that control circadian rhythms. **(C)** In aging neurons, toxic polyglutamine proteins accumulate at PML nuclear bodies. The result of this accumulation is not known in most cases but can lead to the clearance proteins in specific neurodegenerative disorders.

These data present a multifaceted picture of PML expression in the brain. PML is clearly expressed in early development and in aging neurons in the context of neurodegenerative disorders. Its presence in mature, healthy neurons appears to be more complex. A recent review hypothesized that, after development is complete, the adult brain may reacquire PML expression (18). The evidence supports this idea and indicates that PML is expressed in mature neurons under certain conditions, including in the SCN during stages of the circadian cycle, and in response to external stimuli, such as neuronal activity (Figure 1). These findings also suggest that it has an important functional role the brain.

## PML Function in the Brain

Promyelocytic leukemia has several distinct functions in the nervous system, including neuronal development, circadian rhythm regulation, synaptic plasticity, and the neuronal response to toxic proteins that cause neurodegenerative disorders. PML is most commonly studied in the context of dividing cells, and not surprisingly, one of the first indications of the importance of PML in the brain came from studies of the dividing neural progenitor cells (NPCs) during brain development (4). Regad et al. found that NPCs from PML knockout mice (PML^−/−^ mice) have reduced differentiation into neurons that results in a decrease in thickness of the cortex wall. In NPCs, PML regulates the distribution of the retinoblastoma protein and the protein phosphatase 1α. This triggers retinoblastoma protein dephosphorylation, which regulates the transition between radial glial cells and basal progenitor cells.

In addition to its role in brain development, PML also has a role in mature neurons. As described above, PML is expressed in the SCN, which regulates the circadian clock (7). In the SCN, it interacts with the clock regulator PER2 and enhances transcription of circadian clock genes. SIRT1 deacetylates PML at K487, which regulates the interaction of PML and PER2. In PML^−/−^ mice, the expression of *Per2* and other clock regulators is disrupted, and the circadian period has reduced precision and stability. The developmental deficits in PML^−/−^ mice can have confounding effects for studies of the adult brain in these mice. However, in this case, evidence that does not rely on the PML knockout mice also supports a functional role for PML in the adult brain in regulating circadian rhythms. For example, the authors demonstrate that SIRT1 deacetylates PML by SIRT1 knockdown and show that PML regulates transcription of clock genes through PER2 by PML overexpression in combination with PER2 knockout mice.

Increasing evidence also supports a role for PML in regulating synaptic plasticity in the brain. Bloomer et al. found that PML interacts with the protein Arc (19). Arc is crucial for learning and memory and is involved in multiple forms of synaptic plasticity, the molecular mechanisms that underlie memory formation (20, 21). We found that PML is regulated by neuronal activity and required for the neuronal response to increased activity. The protein Arc mediates the activity-induced increase in PML, and PML is required for Arc’s effects on transcription of genes that control synaptic strength (9). Through this mechanism, PML is involved in homeostatic plasticity, which allows neurons to maintain the same firing rate in the presence of persistent changes in activity and is crucial to their ability to store information. In addition to this new evidence, a further suggestion of PML’s involvement in plasticity comes from its known interacting partner death-associated protein 6 (DAXX). DAXX acts as a chaperone for the histone H3.3 variant and promotes H3.3 loading at regulatory regions of activity-dependent genes in response to neuronal stimulation (22). The role of PML in this process is not yet known, but could be another aspect of PML’s involvement in regulating the neuronal response to activity in the context of synaptic plasticity.

If PML is involved in regulating the cellular plasticity mechanism that mediates memory formation, PML^−/−^ mice should have deficits in learning and memory. In fact, PML^−/−^ mice have several phenotypes relevant to cognitive function (8). They have deficits in conditioned fear learning and spatial memory measured by fear conditioning and Morris water maze tests. In addition, they display deficient long-term depression and, depending on the induction protocol used, have an enhanced response to long-term potentiation induction. However, it is not yet known if these deficits are due to PML function in mature neurons or to developmental problems in PML^−/−^ mouse brains. In addition, PML^−/−^ mice show decreased anxiety-related responses, which contribute to some of the learning phenotypes.

In addition to these functions in mediating the neuronal response to activity in healthy neurons, PML is also involved in the response to toxic proteins that cause neurodegenerative disorders. As described above, many such polyglutamine proteins colocalize with PML or result in disruption or rearrangement of PML nuclear bodies. However, this alone is not evidence of PML’s involvement in these disorders. In most cases, it is not clear whether the accumulation of proteins at PML nuclear bodies and rearrangement of the structures cause the neuronal dysfunction, are a beneficial coping response to the toxic protein, or are inconsequential to the disease. For example, PML nuclear bodies contain protein degradation machinery, and polyglutamine proteins may be targeted for degradation as the cell tries to clear the toxic protein. Colocalization of PML and polyglutamine proteins may result as the highly expressed proteins accumulation at sights of protein degradation machinery, regardless of whether PML is effective in the clearance aggregated proteins. However, in at least one case, PML induction appears to mitigate neurodegeneration. In a cell model of spinocerebellar ataxia-7, PML degrades mutant ataxin-7 (17). In a mouse model of the disease, increasing PML nuclear body formation with interferon beta resulted in increased clearance of ataxin-7 and improved the animals performance on behavioral tests (11).

In summary, PML expression in the brain is regulated by various enzymes and external signals, and PML has several important functions in neurons and neuronal precursors. PML carries out its functions by regulating subcellular distribution of critical proteins in neuronal development, transcriptional regulation in circadian rhythms and synaptic plasticity, and protein clearance of toxic proteins (Figure 1). However, clearly much about PML regulation and function in the brain is still unknown.

## Future Directions

While PML was initially studied in the context of cancer, it clearly has important functions in other cell types such as neurons. A better understanding of the role of PML in non-dividing cell types such as neurons will allow for a more complete picture of the wide range of PML’s functions and may even help provide insight into the functions that are disrupted when PML expression is misregulated in certain cancers. It will also be important to understand PML function in the brain if new approaches to cancer treatment focus on activation of PML (23).

Multiple publications demonstrated that PML has important functions in the brain but clearly much is left to be discovered about PML nuclear bodies in neurons. Probably the least understood area is the mechanisms through which PML carries out its proposed functions. PML nuclear bodies regulate a variety of nuclear functions involved in everything from transcriptional regulation and protein degradation to RNA processing. This makes it difficult to assign a specific mechanism through which PML nuclear bodies carry out their functions. In most of the publications described in this review, it is not yet understood how PML is actually mediating the neuronal processes it regulates. In several cases (such as in circadian rhythms and plasticity), PML appears to regulate transcription. But how it does is not clear. We hypothesized that PML regulates transcription through CREB binding protein (CBP), one of its major binding partners. CBP and CREB are critical proteins in neuronal function, so a role for PML in regulating or degrading CBP would have important consequences for neurons (24, 25). A new area for investigation will involve determining how PML is able to affect transcription in neurons.

Second, it will ultimately be necessary to determine if the deficits in PML^−/−^ mice are due to the developmental problems in the brains of these mice or are because of PML’s actions in mature neurons. Studies of learning deficits in the knockout mice are intriguing but cannot definitively be attributed to a function of PML in development or in regulating plasticity in mature neurons. A conditional PML knockout or knockdown model that only disrupts PML expression in mature neurons will be beneficial in answering these questions.

Finally, the role of PML in disease is unclear. While PML appears to colocalize with many polyglutamine proteins that cause neuronal dysfunction, in most cases it is not clear what the effects of this are. PML has a large number of protein binding partners, and the nuclear bodies contain protein degradation machinery. Because of this, many proteins that are expressed in the nucleus colocalize with some component of these nuclear structures or are targeted there for degradation. In the case of highly expressed polyglutamine proteins, many of which aggregate and overload protein degradation machinery, it is not surprising that such aggregates might accumulate at these structures after being targeted for degradation. While the disruption of the PML body in many of these disorders is intriguing, it is too soon to conclude that PML plays an important role in these diseases. Future studies will need to make use of PML^−/−^ mice or PML knockdown as well as PML induction in order to conclude that PML is either beneficial to the disorder or involved in the resulting neuronal dysfunction.

## Conclusion

From the data described here, we conclude that PML is expressed in at least some regions of the brain through the lifetime of a neuron, from development to death. PML expression is also clearly regulated in neurons both by external signals and intracellular proteins. As in other cell types, PML appears to have multiple functions in neurons and can regulate transcription in the context of plasticity and circadian rhythms and protein degradation in response to toxic proteins. However, much is still unknown about PML in the brain, and we expect this will be an area of fascinating new research in the future.

## Conflict of Interest Statement

The authors declare that the research was conducted in the absence of any commercial or financial relationships that could be construed as a potential conflict of interest.
